# Recent Trends in Covalent and Metal Organic Frameworks for Biomedical Applications

**DOI:** 10.3390/nano8110916

**Published:** 2018-11-07

**Authors:** Georges Chedid, Ali Yassin

**Affiliations:** 1 Gilbert and Rose-Marie Chagoury School of Medicine, Lebanese American University, P.O. Box 36, Byblos, Lebanon; georges.chedid01@lau.edu; 2 School of Arts and Sciences, Lebanese American University LAU, P.O. Box 36, Byblos, Lebanon

**Keywords:** covalent organic frameworks (COFs), metal organic frameworks (MOFs), nanomaterials, biomedicine, drug delivery systems

## Abstract

Materials science has seen a great deal of advancement and development. The discovery of new types of materials sparked the study of their properties followed by applications ranging from separation, catalysis, optoelectronics, sensing, drug delivery and biomedicine, and many other uses in different fields of science. Metal organic frameworks (MOFs) and covalent organic frameworks (COFs) are a relatively new type of materials with high surface areas and permanent porosity that show great promise for such applications. The current study aims at presenting the recent work achieved in COFs and MOFs for biomedical applications, and to examine some challenges and future directions which the field may take. The paper herein surveys their synthesis, and their use as Drug Delivery Systems (DDS), in non-drug delivery therapeutics and for biosensing and diagnostics.

## 1. Introduction

Supramolecular chemistry, or “chemistry beyond the molecule”, has presented a new paradigm for molecular engineers [1,2]—an achievement that merited the 1987 Nobel Prize [3]. Supramolecular chemistry has allowed researchers to design molecules with custom properties, like chirality [4]. The development of supramolecular chemistry has opened new subfields of nanoscience. Nanoparticles such as liposomes, micelles, and other small polymers have already been designed for biomedical applications, such as drug delivery [5,6,7]. Covalent organic frameworks (COFs) and their metal organic framework (MOF) cousins are exciting, medically relevant nanomaterials made possible by this new chemistry.

The first reported COFs were the boron-ring-based COF-1 and COF-5, synthesized by Côté and coworkers in 2005 [4]. A COF is a two-dimensional (2D) or three-dimensional (3D) structure with a backbone of light atoms [8], which are held together by strong covalent bonds [9]. Desirable properties of COFs include regular porosity, crystallinity, and high Brunauer-Emmett-Teller surface area (*S*_BET_), which where demonstrated in Côté’s groundbreaking work [4,10]; other properties include well-defined pore aperture, ordered channel structure, low density, stability, mechanical strength, and a wide band gap [8,11,12].

Numerous applications of COFs have been proposed, including separation, catalysis, optoelectronics, sensing, as well as drug delivery and other biomedical uses. COFs have been demonstrated as agents for gas and small-molecule separation, adsorption, and detection [8]. They can be made selective to particular gasses, such as CO_2_, CH_4_, and H_2_; and they may be mounted on a solid support like ceramic or Al_2_O_3_ [8]. They have been designed for selectivity towards trace amounts of different analytes such as NH_3_ [13,14], food dies, and Uranium [8]. Catalytic COFs have been synthesized and demonstrated, some in association with transition metals like palladium [8]; as efficient, recyclable H_2_ photocatalysts [15]; others still as chiral catalysts—thanks to post-synthetic modification (PSM) [16,17]. COFs have also been developed for optoelectronics [18]. Materials with high charge-carrier mobility have been developed, and they are usually based on porphyrin, fullerene, and other groups [19,20]. COFs for improved lithium batteries have also been designed [8,20]; and capacitors with integrated COF electrodes have remained functional over thousands of cycles. COF sensors can detect albumin, explosives, mercury, and other substances [8], and COF films may prove particularly useful as biosensors and drug delivery systems (DDS). Research has already yielded stimulus-sensitive COF materials [21], boasting properties like high drug loading capacity, good release profile, low inherent cytotoxicity, and effective release of drug guests such as 5-fluorouracil (5-FU), ibuprofen (IBU), and doxorubicin (DOX) [8,22,23,24].

Metal organic frameworks were first reported in 1989 by Hoskins [25,26]. In general, a MOF is a crystalline network of a single metal ion or a metal cluster connected to multidentate organic linkers, which are themselves linked by strong covalent bonds [2,27]. Some of the remarkable properties of MOFs include high *S*_BET_ [28]—up to 7000 m^2^·g^−1^ [29]; flexibility and low density [27]; tunable porosity, and diversity in metals and linkers [28]. Some MOFs can even reversibly change their pores upon receiving external stimuli [27].

Many applications of MOFs have been demonstrated, from catalysis to separation, to sensing, and back. They have been validated as heterogeneous catalysts, in which either the metal center or accessible organic units function as the catalytic unit [27,28]. Other applications incorporate separate catalytic species into the framework, a technique already demonstrated in mesoporous silica impregnated with enzymes [30]. MOFs have been designed to selectively store and separate industrially important substances like ethyne and carbon dioxide [28]; they have been synthesized for air quality control, proton conduction, solar energy, and liquid-phase separation applications [27,28]. Sensing MOFs have been developed, capable of colorimetric or optical detection of substances like explosives, aflatoxin, antibiotics, and some inorganic species down to ppb concentration; MOFs have even been developed for luminescent detection of substances like salicylaldehyde [31]. Others have been designed for selective sensing of biomolecules such as ribonucleosides [32]. Finally, a plethora of MOFs have been developed for potential drug delivery applications.

**Figure 1:**
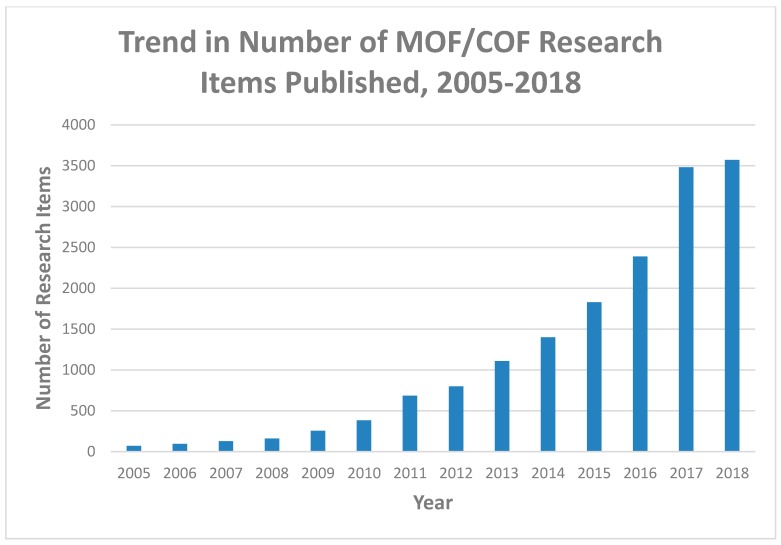
Growth in research interest of covalent organic frameworks (COFs) and metal organic frameworks (MOFs) for biomedical applications. Based on Google Scholar data [33], retrieved 22 August 2018. Google Scholar search term: ‘“metal organic framework” OR “covalent organic framework” biomed OR drug’.

Work on COFs and MOFs for biomedicine has been far from stagnant. Indeed, a glance (Figure 1) at the number of papers published in this area since 2005—the year COFs were discovered—reveals an exponential growth of interest.

Much of this recent work has been characterized by genuine scientific progress coupled with some emerging drawbacks [34,35,36,37]. Recent advances include the use of modulators to improve crystallinity of frameworks; permanent porosity; and the linking of proteins inside COFs and MOFs [9]. Progress has been made toward solving the “crystallization problem” [38], which historically has hindered the construction of crystalline extended structures with metal-charged-ligand or nonmetal-nonmetal bond. On the other hand, disadvantages—particularly for MOFs—abound. These frameworks are frequently synthesized using toxic metals, linkers, and/or solvents [26], and may be chemically or thermally unstable [27]; certain first-generation MOFs have been known to collapse upon removal of their guest molecules [27].

Nevertheless, nanocarriers like COFs and MOFs hold exceptional promise with respect to four basic facets of biomedicine, according to Horcajada [26]:

1. Cell- or tissue-specific targeted drug delivery,
2. Transport of drugs across barriers,
3. Delivery to intracellular sites, and
4. Visualization of drug delivery sites, e.g., theranostics, the fusion of diagnosis and treatment [39].

Furthermore, properties particular to COFs and MOFs make them especially suitable for biomedicine, including their large surface areas; biodegradable and biocompatible structures [40,41,42]; newly described functionalization of COF and MOF scaffolds [43]; and the amenity of both the inner and outer surfaces of these frameworks to functionalization, thus uniting diagnosis and treatment [36,37,44,45].

In view of the absence of a recent review of COFs and MOFs for biomedical applications, this study aims to survey current work, and to examine some challenges and future directions the field may take.

## 2. COFs and MOFs’ Place in the World of Nanomaterials

COFs and MOFs share some characteristics with their nanomaterial cousins. Chief among these relatives are the 2D nanomaterials, which are mainly inspired by graphene and its high surface area, high electron mobility, and high thermal conductivity [12]. Members of this family include black phosphorus, transition metal nanomaterials, graphitic nitride, and organic polymers—COFs, MOFs, and polypeptides. These materials are largely biocompatible, and they may be functionalized with nucleic acids to yield contrast agents for imaging. They can be incorporated into quantum dots, and some undergo fluorescence quenching. Advantages of the organic polymers—particularly COFs—include characteristics like low weight, flexibility, tenability, and adaptability.

As previously mentioned, COFs have been identified as a group of crystalline 2D or 3D structures [8] with backbones of light atoms, held together by strong covalent bonds [9]. COFs belong to the porous organic framework (POF) family of supramolecular structures, whose other members include polymers of intrinsic microporosity (PIMs); conjugated microporous polymers (CMPs); hyper cross-linked polymers (HCPs); porous aromatic frameworks (PAFs) [8]; microporous polymer networks (MPNs) [46]; and liquids with permanent porosity (LPPs). LPPs, first reported in 2015, are a class of free-flowing liquid with bulk properties determined by porosity [47]. Nanoparticles from these related families are the subject of intense research, from improved synthesis to novel applications. Trunk and coworkers, for example, created a new synthesis protocol—without a metal catalyst—for high-surface-area CMPs [46]. Yassin and coworkers demonstrated selective, high uptake (approx. 100 mg·g^−1^) of CO_2(g)_ in reusable MPNs, and showed that isosteric heats of absorption are proportional to the amount of functional group present [48]. Interestingly, given the amorphic nature of MPNs, this family showed high accessible surface area, robustness, and stability, even when compared to COFs [46].

COFs afford a number of highly desirable properties, including porosity, crystallinity, well-defined pore aperture, ordered channel structure, large surface area, low density, stability, mechanical strength, and a wide band gap [8,11,12]. COFs also allow their creators to fine-tune particular properties [11,12]. Certain covalent bonds are commonly used in the creation of COFs, such as boron ester B-O bonds [8], Schiff base C=N bonds [8,49], B-N bonds, N-N bonds, B-Si-O bonds [49], and C-C irreversible linkage [50].

Metal organic frameworks, on the other hand, are crystalline networks of a single metal ion (or a metal cluster) connected to multidentate organic linkers that are tied together by strong covalent bonds [2,27]. MOFs are porous coordination polymers with highly ordered structures, and they are commonly observed as metal-oxide units tied to organic linkers via metal-carbonyl bonds [9]. When choosing metals and linkers for biomedical MOFs, researchers must strike sensitive balances in their selection criteria: degradation kinetics, biodistribution, accumulation and excretion, and the MOF’s specific application [2]. MOFs boast inherent desirable properties like high *S*_BET_ [28] (up to 7000 m^2^·g^−1^ [29]), tunable porosity, wide diversity in metals and linkers [28], low density, and flexibility, and some 3rd-generation MOFs can reversibly change pores upon receiving external stimuli [27]. There are some notable disadvantages, however. MOFs may be chemically or thermally unstable; many metals, linkers, and solvents used in their synthesis are toxic, presenting problems for potential use in medicine [26]; and 1st-generation MOFs may collapse upon guest removal. Common MOF families include zinc carboxylate-based MOFs, like MOF-5; copper (II) square paddle wheel, like HKUST-1; zirconium-based MOFs, like UiO-66; trivalent transition metal—carboxylate-based MOFs, like the MIL series, the subject of study as DDS; zeolite imidazolate frameworks, e.g., the ZIF series; and new frontiers like MOF-polymer blends [27]. In addition, anionic silicate organic frameworks (or Si-COFs), which are hexacoordinate silicate frameworks with metal counter-ions like lithium, sodium, and potassium, have been reported [51,52].

More distantly related materials include mesoporous silica [30] and dendritic polymers, which—when modified with polyethylene glycol (PEGylation)—show anti-tumor effects in vitro [53]. PEGylated liposomes, another unrelated nanotechnology, are approved by the American Food and Drug Administration (FDA) for cancer treatment [5]. These medicinal techniques rely on the enhanced permeability and retention (EPR) effect. In other words, a tumor must have well-enough developed blood vessels for nanoparticle effectiveness. A final group of nanomaterial relatives is electro-generated nanostructured materials [54], which are synthesized by electropolymerization; these materials were used to make highly ordered, stable electrodes. They demonstrated high fluorescence yield and Stokes’ shift, and hence high oxidation potential.

## 3. Synthetic Methods

### 3.1. Synthesis of COFs

Thanks to reticular chemistry—the linking together of chemical entities to make frameworks, pioneered by Omar Yaghi [9] and others—synthesis of crystalline open frameworks is now possible. Common synthetic strategies include solvothermal, ionothermal, microwave, mechanothermal, and room-temperature methods, as well as interface synthesis [50]. COF synthetic reactions are generally controlled by changing the solubility of linkers and the amount of water [52]; for imine-linked COFs, the amount of in-situ generated amine may be controlled (and, analogously, the amount of in-situ silicon, for Si-O linked COFs). Some COFs are subjected to PSM to add ligating hooks like amino groups [55]. This technique is essential for COFs in living systems, as demonstrated by the addition of folic acid to COFs for effective cancer-cell targeting [56]. Other techniques include self-templated synthesis, which has been used to make COF spheres via the Ostwald ripening process [57].

Some 2D COFs are produced via exfoliation, the transformation of bulk COF into covalent organic nanosheets (CON) [56,58]. COFs may be unstable in water due to hydrolysis; this problem, along with poor dispersal associated with some COFs, has been largely solved by engineering for keto-enol tautomerization [56]. Self-exfoliation without external stimuli has been reported, which solves some problems associated with exfoliation, such as restacking, difficult syntheses, π-π stacking, and polymer instability [59].

One novel technique described by Tan [60] is an amorphous-to-crystalline transformation, whereby Fe_3_O_4_ nanoclusters are embedded in an amorphous polyimine network whose bonds were reconstructed under thermodynamic control to yield a COF shell. This method allows for retention of certain properties (uniform spherical size and shape) and addition of new ones (controllable COF shell thickness, PEG-modifiable surface, crystallinity and surface area that increase with reaction time). This amorphous-to-crystalline technique negates the inherent difficulty encountered in solvothermal methods when controlling purity, size, or shape of the product COF.

### 3.2. Synthesis of MOFs

Similar techniques have been used to produce MOFs. Early MOFs were made using diffusion techniques, e.g., the infusion of metal salts into solutions containing the linkers. However, this slow and low-yielding technique has been supplanted by modern solvothermal methods [27]. Other methods include grinding, electrochemical, and sonochemical approaches. Post-synthetic modification of MOFs is especially relevant for biomedical applications, as it allows for tweaking of functional groups; fine-tuning of pore size; improved drug performance (hydrophilic MOF pores for drugs with charges opposite to the MOF backbone’s charge). Furthermore, PSM of MOFs allows integration of biomolecules as linkers, and even inclusion of bioactive metal ions [27]. Traditionally, control of MOF synthesis reactions has been achieved by modulating the rate of deprotonation of carboxylic acid linkers [52]. It is believed that in any given MOF synthesis, the networks with the highest symmetry are the networks most likely to result [9].

Recent syntheses, most notably by Shieh and coworkers, employ a de novo strategy to grow MOFs around their biomolecule cargo [61]. This technique may allow efficient, in situ incorporation of biocatalysts or drugs into their MOF delivery systems.

The art of MOF synthesis has fallen victim to “folklore” in the eyes of Cordova [62], namely that MOFs are unstable and expensive to produce; that they have yet to achieve commercial value, and that their synthesis cannot be scaled. It should be noted, however, that certain MOFs are already sold commercially by BASF [62] thus allaying such concerns.

## 4. COFs and MOFs as Drug Delivery Systems

### 4.1. Characteristics of Good Drug Delivery Systems

**Figure 2:**
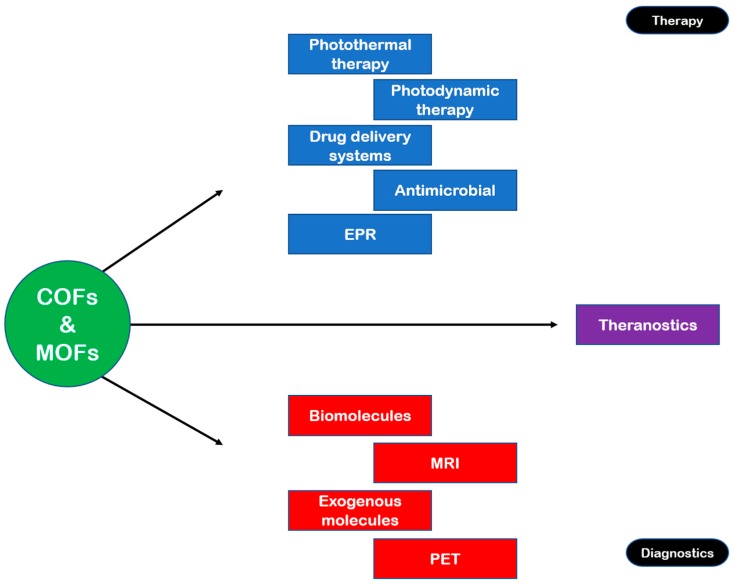
Concept map of COFs and MOFs as therapeutic and diagnostic agents.

Effective drug delivery systems, nanotechnology-based or otherwise, should combine desired characteristics with feasible synthesis and preparation. Such characteristics include high drug loading capacity, sustained release of the drug, local control of in vivo release, and solubility in water [53,56]. Moreover, DDS must be non-toxic and preferably biodegradable; they should be good candidates for surface engineering; and they should have high molecular weights in order to increase circulation time. Commonly reported problems with nanoMOFs and some organic DDS include background leakage of drugs and low pore loading [63]. A table of reported COF and MOF drug-delivery systems, with the corresponding linker, the loading capacity, and time to release load, is presented in Table 1, and a concept map of COFs and MOFs as therapeutic (and diagnostic) agents may be seen in Figure 2.

### 4.2. COFs as Drug Delivery Systems

Despite being a young field, the implementation of COFs in drug delivery has already begun bearing fruit. The first report of COF-DDS came in 2015; this pioneering work by Fang demonstrated effective IBU release from a polyimide covalent organic framework (PI-COF) with drug loading as high as 24 wt % [22]. The system showed a good release profile, tied to the COF’s pore size and geometry. Another early attempt employed PAF-6, an aromatic framework, for IBU delivery [11]. This nontoxic, biodegradable PAF was produced under mild synthetic conditions, without a metal catalyst, and it showed regular, 2D sheets with π-π stacking. Compared to MOFs already studied in the context of drug delivery, such as MIL-53 and MIL-101, PAF-6 showed high release rate and outperformed inorganic nanoparticles such as MCM-41 [11].

More recent attempts have improved the performance and versatility of COF-DDS—even against multidrug-resistant cancers [64]. Tian synthesized a framework in situ for delivery of pemetrexed, a chemotherapeutic drug, in a pH-sensitive fashion [65]. This approach was shown to be effective against MCF-7 breast cancer in vivo and in vitro, and the new DDS overcame multidrug resistance in these cells by leveraging the EPR effect. The framework, taken up by endocytosis, showed better load efficiency than liposomes and had low inherent cytotoxicity. Bai used PI-COFs (endowed with amine groups to hook drug guests) to deliver 5-FU, IBU, and captopril in vitro [23]. This approach showed high drug loading (up to 30 wt %) and good release (days), and the COF pores expanded upon drug loading. Kandambeth used hollow spherical COFs to deliver DOX, a chemotherapeutic agent, with good release profile [57]. Quercetin, another anti-cancer drug, was released by an imine-based COF DDS, showing efficacy against breast cancer cells in vitro (MDA-MB-231) [66]. Liu produced a single-layer, photoresponsive COF, capable of being destroyed under ultraviolet light but recovering upon mild annealing; this technique demonstrated controlled capture and release of a guest molecule (copper phthalocyanine) [21].

Several COFs have been designed to discriminately target drug-delivery sites. Rengaraj synthesized a nano-covalent triazine polymer (CTP) to release DOX in a pH-sensitive manner [24]. After synthesis, the CTP was subjected to ultrasound and filtration to yield nano-CTP. Inherently fluorescent (allowing the researchers to track DDS movement in vitro), this material released DOX at low pH (~4.8) commonly associated with cancer cells (as opposed to physiological pH, ~7.4). When loaded with DOX, the DDS showed higher cytotoxicity against adenocarcinoma cells (Henrietta Lacks, or HeLa cell line) than free DOX. The material was shown to promote cell senescence—substantiated by upregulation of genes p53 and p21, which are implicated in tumor suppression, response to deoxyribonucleotide (DNA) damage, and apoptosis [67,68]. Mitra synthesized COFs for targeted delivery of 5-FU, another chemotherapeutic agent [56]. The COFs were produced using Schiff-base synthesis, followed by exfoliation and ultimately a series of post-synthetic modifications to add targeting ligands. Using amine groups as anchors, the investigators attached folic acid to the COFs to facilitate targeting of breast cancer cells in vitro (MDA-MB-231). This key targeting step lead to receptor-mediated endocytosis of the DDS and, ultimately, apoptosis (programmed cell death). Apoptosis must not be confused with senescence (another goal of anti-cancer drugs), which refers to a cell in a non-dividing inert state.

Attempts have even been made to combine COFs with metal moieties for medicinal purposes. Luo produced a porphyrin-based covalent triazine framework with and without manganese to effectively deliver IBU in vitro—but the material was shown to be amorphous [69]. Still, the covalent nature of the framework, combined with porphyrin’s good metal coordination, allowed for high drug loading (23 wt %), good release profile, porosity, and thermal stability.

### 4.3. MOFs as Drug Delivery Systems

Several properties of MOFs make these nanocarriers ideal for drug delivery. First, the interaction between MOFs and guest molecules is tunable [70,71]. The relationship between MOF hosts and their guests is dynamic and selective; interactions can be predicted using simulation; and some MOFs even retain “memory” of their guests [72,73,74,75]. Second, some MOFs may be loaded with multiple drugs [76,77]. Third, the external surface of MOFs can be functionalized to promote coordinative binding [78], ligand exchange [79], and covalent binding to linking groups [80,81]. Fourth, MOFs can be designed for stimulus-responsive intracellular drug release [82]—both MOF polymers [82,83,84] and MOFs coated with lipid bilayers [85,86] have been reported.

Progress in MOF-based drug delivery has had the advantage of time; research on MOF-DDS was ongoing even as COFs were being discovered. MOFs have been engineered to deliver both endogenous substances and synthetic drugs. One such endogenous substance is nitric oxide (NO), a biologically active signaling molecule that is commonly employed in surgery and dialysis; it is critical for clotting, the nervous system, and the immune system. Highly crystalline copper-tricarboxylate-based MOF HKUST-1 has been shown to absorb and release NO upon contact with water vapor; NO has antibacterial, antiplatelet, and vasodilatory effects [87]. Unfortunately, HKUST-1 showed instability in water (a problem endemic to MOFs) and turned the aqueous solution green within hours of exposure to plasma. Nitric oxide was shown to bind directly to the Cu core in HKUST-1; in fact, Horcajada [26] affirms that “…every single MOF that has open metal sites seems to bind NO to a significant degree”. This suggests potential for MOF-based NO delivery systems, perhaps by coating artificial surfaces (dialysis tubes, artificial valves, stents) with such materials.

Various other MOFs have been exploited for intriguing ends. Iron (III)—based MOFs such as MIL-53, MIL-88A, MIL-88Bt, MIL-89, MIL-100, and MIL-101_NH_2_ have been tested for effective in vivo delivery of anticancer and antiretroviral (HIV) drugs, such as DOX, azidothymidine, and busulfan [40]. Uptake and release of caffeine by MOFs have been studied [88], and platinum-based MOFs have effectively killed human colon carcinoma cells in vitro [27]. The first biodegradable MOF, Fe-based BioMIL-1, was made with organic linkers that themselves functioned as the drug [2,89]. The linker in this pioneering work was nicotinic acid, or niacin, a form of vitamin B, with vasodilating, anti-lipemia, and anti-pellagra properties. This linker, coupled with relatively nontoxic iron, should serve as a model for other MOF-DDS. MOFs with various mixed ligands have been studied for the controlled delivery of IBU and DOX [90].

MOFs have been the subject of theoretical study as well; multiple papers combine in silico studies with traditional wet chemistry. Babarao and coworkers conducted a complete computational analysis of IBU loading and release in a range of mesoporous MOFs and empirically confirmed stronger binding and slower release in MIL-101, as predicted by calculations [91]. The investigators accurately predicted high loading capacities (MIL-101: 1.38 g·g^−1^, MIL-53: 0.22 g·g^−1^ but independent of central metal, e.g., Cr or Fe) using Monte Carlo, molecular dynamics, and density functional theory techniques. They showed that guest molecules administered via a nanoporous vehicle behave differently with respect to normal bulk administration, and they predicted a carboxyl group rearrangement in MIL-101. A newer study by Li analyzed a 3D Cu(II)-carboxylic MOF carrying 5-FU and effective against spinal cord cancer in vitro; the authors accurately predicted the drug loading capacity (17.3 wt %). They also investigated the MOF’s success at avoiding the undesirable burst effect, the rapid release of much of the drug payload [92]. A recent study led by Rojas analyzed the adsorption/desorption kinetics of various drugs (IBU, aspirin) and MOFs (MIL-100, UiO-66, MIL-127) [29]. The authors concluded that drug loading and delivery are influenced primarily by two factors: (1) structure, e.g., framework accessibility and drug volume; and (2) the MOF/drug hydrophobicity/hydrophilicity balance.

Recent studies have yielded MOFs both sensitive to stimuli and capable of targeted drug release. Many syntheses of targeted MOF-DDS rely on post-synthetic modification, e.g., attaching ligands (such as folic acid or special substrate peptides that can bind cancer cells) to the MOF [93,94]. Chen designed a DOX-loaded nanoMOF coated with a nucleic acid hydrogel, sensitive to adenosine triphosphate (ATP), so that the drug was released in the presence of high concentrations of ATP, which is commonly overexpressed in cancer cells [63]. Advantages of hydrogel-coated MOFs over traditional MOFs, exemplified in this study, include higher drug loading, lower leakage, and greater cytotoxicity toward cancer cells. Remarkably, this DDS was selective to ATP alone; that is, other nucleotide triphosphates (GTP, CTP, TTP) did not trigger drug release. This UiO-68—type, Zr-based MOF was effective against breast cancer (MDA-MB-231) in vitro and employed a DNA switch as a release trigger. (A DNA switch, seen in DNA machines, hydrogels, and sensors, is a supramolecular structure that reversibly reconfigures itself in the presence of signals such as pH, enzymes, or light [95].) DNA was linked to the MOF via click chemistry; and the overall DDS showed minimal cytotoxicity toward healthy cells. In a subsequent study, Chen constructed another Zr-based, DOX-loaded nanoMOF, modified with a nucleic acid sequence complementary to a vascular endothelial growth factor (VEGF) aptamer [95]. VEGF is over-expressed in conditions like macular degeneration, diabetic retinopathy, rheumatoid arthritis, bronchial asthma, and diabetes mellitus; an aptamer is a nucleotide or peptide molecule that binds to a specific target molecule. As in the previously mentioned study by the same investigator, a DNA switch was used to trigger the release of DOX in the presence of VEGF. The aptamer was used to target specific receptors (e.g., nucleolin) on cancer cells. This nanoMOF exhibited performance superior to that of SiO_2_, with higher drug loading, better dispersion, and comparable background release.

Hybrid MOFs have begun to appear in drug delivery contexts. Liang [96] produced a core-shell protein@ZIF entity that released DOX at low pH, similar in concept to the pH—triggered drug release investigated by Rengaraj [24] and Tian [65]. This protein@ZIF hybrid, effective against breast cancer (MCF-7) in vitro, consisted of a DOX/Bovine Serum Albumin (BSA) core surrounded by a ZIF-8 shell. In environments with low pH (5.0–6.0), dissociation of the ZIF was triggered, promoting drug release [96]. Impressively, this hybrid showed crystallinity, effectively no leaching of drug, and better biocompatibility than bare ZIF-8. The investigators demonstrated their ability to adjust the hybrid particle’s size by varying the concentration of NaCl during synthesis. MOFs have also been combined with graphene oxide (GO) to form MOF/GO composites for efficient, controlled, and tunable delivery of drugs [97].

MOF-based DDS have generated much interest in the chemical community at large, and MOFs’ utility in other areas of interest to biomedicine has been demonstrated as well.

## 5. Applications: Non-Drug Delivery Therapeutics

### 5.1. Photothermal Therapy

**Figure 3:**
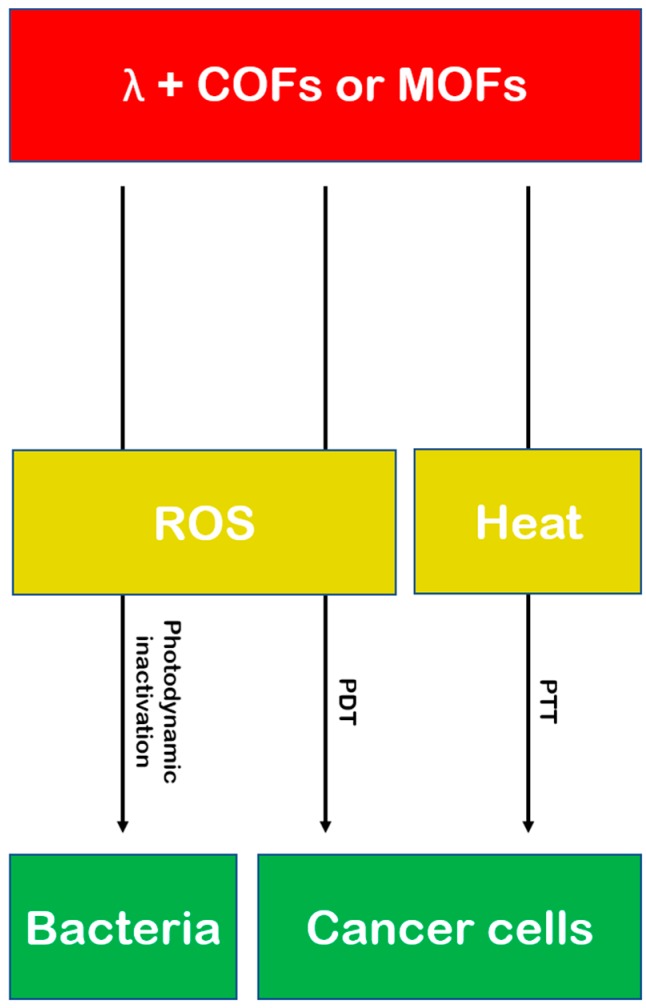
Photodynamic (PDT) and photothermal (PTT) therapy, with COF or MOF agents, against bacteria and cancer cells.

Photothermal therapy is a modern antitumor treatment whereby targeted radiation excites a photosensitizer molecule, which in turn generates heat and kills cancer cells by thermal ablation. This phenomenon has been demonstrated with some success in graphene [60]. Covalent photosensitizers are believed to be advantageous for this technique, as they tend to be biocompatible, efficient photoconverting agents. This technique is outlined in Figure 3 (along with other non-drug delivery therapeutic techniques that involve COFs and MOFs).

Some COFs have been designed with photothermal therapy in mind. Tan used a self-sacrificial template to construct a COF with an integrated Fe_3_O_4_ core and efficient photoconversion ability, allowing for rapid killing of HeLa cells in vitro [103]. The researchers were able to modulate shell thickness and sphere cavity size, and the COF showed low inherent cytotoxicity. Irradiation by near infrared (NIR) laser caused rapid heating—up to 24 °C within six minutes—and the authors suggested targeted delivery using a magnetic field in vivo, owing to the magnetic nature of the Fe_3_O_4_ cores. A subsequent study by the same author [60] was the first reported demonstration of an imine-based COF with photoconversion ability (again associated with a Fe_3_O_4_ core), thanks in part to the COF’s layered π-π stacking. Although a large, quick temperature change was observed, and the COF shell enhanced light absorption, several drawbacks were encountered. The investigators reported difficulty in modulating the COF during growth, due to inconsistent Ostwald ripening.

MOFs for photothermal therapy have been described as well. Photosensitizers can be incorporated into the MOFs’ structure, as reported for fourth-generation MOFs [104]. Wang reported a polymer-MOF (UiO-66) composite for photothermal therapy that was shown to be effective against colon cancer cells in vivo [105]. A recent investigation [39] reported a core-shell structure for synergistic photothermal therapy and chemotherapy, with efficacy both in vitro (breast carcinoma line 4T_1_) and in vivo (90% tumor suppression in mice). A mesoporous ZIF-8 MOF shell was deposited around a single gold nanorod core and loaded with DOX. This hybrid material showed dual sensitivity to low pH and NIR irradiation. This synergistic approach caused much more thorough tumor suppression (~90% suppression) as opposed to ~58% under irradiation alone, and a mere ~30% without NIR irradiation. This new approach presents substantial advantages: high drug loading, stability in physiologic-like media, and biocompatible components with efficient light-to-heat conversion. This chemical-radiative synergy helps overcome drawbacks inherent to other approaches: some organics may release drugs inefficiently, and some inorganics are difficult to synthesize and may not be biocompatible.

### 5.2. Photodynamic Therapy

Another modern anti-tumor treatment is minimally invasive photodynamic therapy: a photosensitizer generates reactive oxygen species (ROS, ^1^O_2_) in response to irradiation, and the ROS go on to kill cancer cells [106]. Photosensitizers must have high quantum yields, long lifetimes, and good Stokes’ shifts. Typical photosensitizers, like porphyrin, present certain problems related to their hydrophobic nature and π-π interaction, ultimately leading to aggregation and inefficient ROS generation.

COFs, on the other hand, are theorized to be excellent photosensitizers. They are covalent networks with periodicity—a trait not shared with CMP, CTF, and POP molecules. In addition, COFs possess desirable photosensitizer characteristics that MOFs do not: large, accessible pores, exceptionally well-ordered structure, low density, and thermal stability [107].

A few COFs have been designed with photodynamic therapy in mind. Using an imine-condensation reaction, Lin synthesized a porphyrin-based COF (3D-Por-COF) to effectively generate ROS [108]. While the integration of photoelectric moieties into COFs had thus far proven elusive, investigators nevertheless reported effective ^1^O generation under photoirradiation and were able to modulate the COF’s properties by metalating the porphyrin rings. ROS generation was effective over multiple cycles, and the framework structure was maintained throughout. Bhanja created a novel N-containing COF (EDTFP-1) for photodynamic therapy [107]. The ROS-generating ability of this material was tested with a number of cell lines in vitro over a range of pH values, effectively triggering apoptosis in cancer cells via a p53-mediated pathway.

MOFs, too, have found some success as photodynamic therapy agents. A composite MOF/imine-based organic polymer (UNM) with a core-shell structure was designed by Zheng for ROS generation [106]. UNM boasted porosity and high surface area, but the covalent shell was shown to be amorphous. ROS generation was inhibited by vitamin C, a recognized ROS scavenger. The material was shown to be effective against HeLa cancer cells in vitro with uptake via ATP-mediated endocytosis; interestingly, the apoptosis was concluded to be dose-dependent, e.g., governed by radiation power and time. Other MOFs—mostly based on porphyrin—have been designed for photodynamic therapy. A Zr-porphyrin MOF known as PCN-222 was originally designed for general biocatalysis [109] and has recently been adapted for anticancer photodynamic therapy [110]. One group was able to modulate the size of its porphyrin-based MOF for effective, targeted photodynamic therapy [111]; another group decorated a MOF’s surface to improve stability and photodynamic activity against cervical cancer cells [112]. Other groups have designed chlorin-based MOFs for photodynamic therapy [113] as well as photodynamically-active MOFs that target mitochondria [114].

Two groups have devised novel ways to employ MOFs for photodynamic therapy in hypoxic environments; using two distinct methods, they equipped MOFs with chemical machinery capable of generating oxygen. Li embedded catalase (an enzyme capable of converting hydrogen peroxide into oxygen) into a photoactive MOF, thus ensuring that photodynamic activity could continue in oxygen-poor tissues in vivo [115]. Impressively, another enzyme was also included to effectively starve cancer cells. Recently, Lan reported the inclusion of Fe_3_O clusters in a MOF to convert endogenous H_2_O_2_ into O_2_ for photodynamic therapy [116].

### 5.3. Adsorption of Heavy Metals

Heavy metals are implicated in a variety of health problems, especially neurologic diseases. Although many studies of heavy metal uptake in COFs and MOFs have focused on environmental pollution and water remediation, it possible that these materials will one day serve as antidotes to toxic metal poisoning in humans, as well. COFs offer some tantalizing characteristics needed for metal adsorption, like the ability to graft coordination groups to their porous base; fast, selective, high-capacity adsorption; and regular pores, making them preferable to molecular sieves [117]. However, not all coordination groups are compatible with current synthetic methods.

COFs promise brilliant performance in mercury removal from aqueous media. Mercury is a common pollutant, implicated in neurologic afflictions like Minamata disease, which leads to convulsions and death [118]. The use of porous adsorbents is cheaper and simpler than chemical methods of trapping Hg. COF-LZU8, a pioneering material, combined detection of mercury (fluorescence) with removal. This material, developed by Ding [119] employed a thioether group as an Hg^2+^ receptor–fluorescence quencher. Sensitive to very low concentrations (25.0 ppb), recyclable, and selective to Hg^2+^, COF-LZU8 set a high bar for this subfield despite some problems with dispersion. More recently, Huang made TAPB-MBTTPA-COF to selectively trap Hg (II) from aqueous media via thioester ligation [118]. This imine-linked COF contained high sulfur content (15.5 wt %) and remained stable in both acidic and basic media. The COF captured Hg—up to 734 mg·g^−1^, more efficiently than COF-LZU8. TAPB-MBTTTPA-COF was recyclable, sensitive (10 ppm), and selective to Hg^2+^. Another paper, by Sun [117], described COF-V’s effective removal of both Hg^2+^ (up to 1350 mg·g^−1^) and Hg^0^ (up to 863 mg·g^−1^) from gaseous and aqueous media via exploitation of Hg-π interaction. This recyclable COF contained a vinyl moiety to allow for straightforward PSM.

MOFs have shown some utility in absorbing heavy metals. Some frameworks have been designed to efficiently remove lead and malachite green from water [120,121]. Arsenic removal by MOFs has been recently described [122]; stable AUBM-1 was effective over a wide pH range (1–14) and demonstrated high adsorption of As from water (up to 103.1 mg·g^−1^ at neutral pH), thus outperforming commercially available adsorbents (<100 mg·g^−1^). Arsenic is implicated in skin damage, circulatory problems, and an elevated cancer risk.

### 5.4. Antimicrobial Activity

In an age of increasing antibiotic resistance, development of alternative anti-bacterial agents is critical. Some groups have leveraged the sensitivity of microbes to reactive oxygen species, an approach known as photodynamic inactivation; other groups have used other approaches. Many groups have reported greater success against Gram-positive bacteria than Gram-negative bacteria; the latter are covered by a thick glycocalyx, which is made of negatively charged (and toxic) lipopolysaccharide, which enhances resistance to photodynamic inactivation [123,124,125]. Much work has been reported on nanomaterials other than COFs [123,126,127] and MOFs [128,129,130,131,132,133] for photodynamic inactivation.

A few examples of COFs for photodynamic inactivation of microbes have been reported. Triazine-based COFs have been designed by Liu [134] for this goal. These COFs (COF-SDU1 and COFs-Trif-Benz) were effective against *Escherichia coli* (gram-negative) and *Staphylococcus aureus* (gram-positive). The most prominent example of COF-based photodynamic inactivation was recently reported by Hynek and coworkers [135]. They designed a 3D porphyrin-based COF that effectively generated singlet oxygen under visible-light irradiation at intensities as low as 1 mW cm^−2^, and killed resistant *Enterococcus faecalis* (a gram-positive facultative anaerobe) as well as *Pseudomonas aeruginosa* (a gram-negative aerobe). Such bacteria are challenging; infectious-disease specialists must balance—on one hand—the urgency of treating infected patients and keeping hospitals clean—against the looming threat of antibiotic resistance, on the other. It is remarkable that this COF was effective against both gram-positive and gram-negative bugs under visible-light wavelengths. Antimicrobial surface coatings based on this COF may prove quite useful when incorporated into medical devices, bandages, and so on.

So far, not many MOFs have been designed specifically for photodynamic inactivation. A new surface-anchored, porphyrin-based MOF, dubbed “SURMOF”, demonstrated antimicrobial activity via ROS against *E. coli* under visible light [136]. Potentially, this MOF could be deployed as a thin film and guest molecules such as antibiotics could be incorporated.

Other approaches do not involve photodynamic inactivation. One solution might be 4-4’-bipyrazoyl-Ag discs, which are effective against three different strains of bacteria, including *Staphylococcus aureus* [27]. In 2016, Mitra [59] demonstrated a COF with incorporated guanidium ions for antimicrobial activity. This COF was synthesized via exfoliation without external stimuli, and was effective against a variety of bacteria, both gram positive and gram negative. The authors surmised that the intrinsic charge on the COF backbone facilitated the entry of the COF into negatively charged bacterial cell membranes, causing membrane rupture. As a side note, some researchers have reported success with photodynamic inactivation based on modified silica gel [137].

### 5.5. Other Uses

COFs and MOFs have been prepared for various other biomedical applications. Lohse synthesized an imine-based 2D COF for lactic acid absorption, employing PSM to create appropriate hooks without interfering with COF formation or π-π stacking [55]. Kandambeth made a hollow spherical COF to immobilize the protein trypsin, with potential applications in industry and the health sciences, possibly as a biosensor [57]. Targeting of nanomaterials was explored by ligating nanoMOFs to cyclodextrin [27], thus allowing these MOFs to bind specific receptors or even circumvent the immune system. MOFs have been synthesized with the use of cell walls (fungal and bacterial) as supports [138], facilitating size-selective, slow release of guests. Finally, a recent study employed a UiO-66 type Zr-based MOF for absorption of the nonsteroidal anti-inflammatory drug (NSAID) ketorolac tromethamine from water [139]. This reusable MOF, interestingly, used linkers sourced from recycled polyethylene terephthalate plastic bottles, paving the way for future green synthesis of frameworks.

## 6. Applications: Biosensing and Diagnostics

### 6.1. A Brief Survey of Current Techniques

Modern imaging is an integral part of 21st-century medicine. Common advanced imaging techniques include computed tomography (CT), magnetic resonance imaging (MRI), positron emission tomography (PET), ultrasound, and optical imaging. Several of these techniques require contrast agents, which may be ingested by the patient [140]. Approaches used when applying nanotechnology to diagnosis fall into two broad categories: electrochemical biosensing/imaging, and optical biosensing/imaging [12].

### 6.2. Considerations in Designing Materials for Biosensing and Diagnosis

Materials scientists must be mindful of certain constraints when designing contrast agents and other chemicals for imaging. Some fluorescent frameworks employ “turn-on” fluorescence, the emission of light upon reception of stimuli; others are of the “turn-off” type [140]. The path a nanoparticle takes through the body is dictated by its design: primarily its size (ideally 60–100 nm), shape (rods are preferable to spheres), and surface chemistry. Additional considerations apply for imaging and diagnosis of cancer. Scientists must consider a tumor’s microenvironment—vascular, deep tissue, or circulating—which affects imaging options. In addition, the EPR effect desired for DDS opposes the design requirements for contrast agents. Whereas the EPR effect relies on accumulation of chemical agents near tumors, accurate imaging requires even distribution [140].

The ideal contrast agent for modern imaging should possess three fundamental properties. First, the agent should have a high rate of margination, that is, the ability of a nanoparticle to escape the blood flow and move toward the blood vessel wall. Larger particles tend to rely on convection; smaller particles, on diffusion. Second, such agents should have strong binding avidity to tumors for accurate visualization. Third, they should be rapidly internalized into cells and tissues.

Reliable, accurate diagnosis is critical, particularly for tumors. It is widely known that early detection and treatment of tumors, generally when they are less than a few millimeters in diameter, correlates with better survival. Blood vessels in young tumors differ greatly from those in metastasized, advanced tumors; younger tumors respond better to targeting, suffer less fluid leakage, and overexpress certain receptors, hence facilitating targeting by imaging and antitumor agents [140].

### 6.3. COFs for Biosensing and Diagnostics

To date, relatively few studies on COFs as biosensing agents have been conducted. Nevertheless, trends in the literature have begun to emerge [12]: luminescence is stronger in 2D than bulk COFs, and exfoliation from 3D to 2D is a valuable synthetic tool in this area. Two-dimensional porous frameworks show good potential [49] thanks to their large, rigid structure with π-π conjugation, strong fluorescence, spatial selectivity, and well-defined, regular pores. Wan reported the first luminescent, semiconducting COF in 2008 [18]. This mesoporous, arene-based, belt-shaped COF set a high bar for subsequent investigations with its high quantum yield (~60%), good on/off switching performance, and characterization as a p-type semiconductor.

Newer work has set new standards. Wang designed an imine-linked COF, layered on a silicon substrate, for DNA detection by measuring the change in impedance [141]. Peng made a 2D imine-linked COF as a selective and sensitive DNA detector [58]. Produced via exfoliation of bulk COFs, this material fluoresced upon encountering specific target DNA, which triggered hybridization between hairpin DNA ligated to the COF. Interestingly, this was the first reported observation of COF building blocks under the transmission electron microscope. More recently, Kong reported the in-situ synthesis of imine-based COF LZU1 for electrochemical detection and separation of amino acids and NSAIDs [142]. The COF was layered in a column for open-tubular capillary electrochromatography to improve its performance. Wang [49] demonstrated PI-COFs for Fe^3+^ detection via metal-ion-induced fluorescence quenching. Lin used a relatively rare 3D COF containing photoelectric units [143]. This material effectively detected picric acid, an explosive, via turn-off fluorescence. This approach leveraged the advantages of 3D frameworks in such an application, e.g., high specific surface area, low density, and multitude of open sites. Wan [144] developed an imine-linked COF for photocurrent generation, similar to the endeavor mentioned in (Wan, 2008) [18]. Finally, Li designed an imine-linked COF to detect the biomolecules DNA and ATP via turn-on fluorescence [145]. The investigators designed this COF, stable in human serum, as a multi-function sensor. In addition, they were able to distinguish single-base-pair mismatches in target DNA, potentially allowing for detection of mutated DNA (a root cause of cancer). The COF’s sensitivity to ATP may yet prove useful for tumor detection.

### 6.4. MOFs for Biosensing and Diagnostics

MOFs, like their COF relatives, offer unprecedented flexibility in the design of targeted biosensing/diagnostic agents [146]. MOFs may be used to position catalysts and magnets [9]; a 2009 study [147] demonstrated homogenous inclusion of iron into a graphitic nitride network for generation of H_2_ and activation of CO_2_. It is possible to engineer MOFs based on enzymes, such as ferritin, and MOFs have demonstrated utility as films (the likes of which are essential for biosensor design) [9]. MOFs have been studied for use with MRI [140,148] and PET [149]. A recent study reported the synthesis of graphene-MOF composites for enantioselective capture of drug intermediates in a magnetic field [150]. In light of the relatively scant quantity of work performed on COFs or MOFs as films, this area merits increased attention.

### 6.5. Theranostics

Theranostics means combining diagnosis and treatment; this is an area in which nanocarriers—especially MOFs, and to a greater extent than COFs—have shown exceptional promise [151]. This approach allows clinicians to accurately target tumors or other areas of interest, thereby ensuring thorough treatment and lessening the required effective dose. For example, Zhao reported a combined magnetic resonance-contrast and DOX-carrying MOF that lead to better therapeutic outcomes than free DOX alone [152]. Some groups have created core-shell theranostic MOFs [153], fluorescent, trackable MOF DDS [154], and targeted, MRI-trackable MOF DDS [93]. Other advances include imaging-trackable MOFs that can be employed for photodynamic therapy [155,156] as well as photothermal therapy [157]. One group even combined controllable drug delivery with MRI and photothermal therapy in a single MOF system [158]. However, it appears that not much work on COF-based theranostics has been reported.

## 7. Future Advances and Obstacles

Despite COFs’ and MOFs’ remarkable performance in the laboratory, turning these frameworks into safe and economical therapeutic and diagnostic agents is expensive, fraught with regulatory hurdles, and difficult from a technical perspective. In general, these problems characterize many attempts to bring new drugs and medical tools to the market.

### 7.1. Regulatory Difficulty

Any substance designed for use in medicine must demonstrate safety and efficacy in rigorous clinical trials before being approved for use on patients. In the United States, the Food and Drug Administration (FDA) is the regulatory body responsible for such approvals.

The FDA upholds rigorous standards and screens new products for safety. A higher margin of safety is required for imaging substances than for drugs, since the former may be administered to healthy individuals [140]. The components of nanotechnology—the various linkers and monomers discussed previously—must conform to published FDA lists, including the Generally Regarded as Safe list, and the Everything Added to Food list [2]. Unfortunately, there is inconsistency in regulatory practices and risk tolerance between different countries; as early as 2015, for example, cocrystal treatment was available and approved in Japan, well before European or American approval [2].

### 7.2. Problems in Translating In Vitro/Small Animal Results into Clinical Results

The papers mentioned in this review largely reported in vitro studies and, in some cases, small animal trials. Unfortunately, it is inherently difficult to translate such studies into real-patient results. In vitro studies neglect hemodynamics and tumor microenvironments, for example [140]. Many experiments are not carried out in physiological media [91].

New technology, like COFs and MOFs, must demonstrate profound effectiveness, not mere sophistication and complexity; indeed, some new nanotechnologies perform worse than traditional treatments [5]. In the early 2010s, for example, thermo-sensitive liposomes found success in mouse trials but failed to demonstrate effectiveness at the subsequent clinical trial stage. Ultimately, medicinal outcomes depend on individual physicians’ and patients’ goals [5].

### 7.3. Existing Nanotechnology Based on Old Principles

Two examples of nanotechnology, based on classic chemical principles, have found resounding success in the clinic: doxil and abraxane. Doxil, a PEGylated liposome, delivers DOX as effectively as the free drug itself but with a higher margin of safety for the patient [5]. Liposomes have been known to the chemical community for some 60 years; PEG, for 40. Work on PEGylated liposome began in the 1980s, but the product was not granted FDA approval until 1995, an unfortunate but typical lag time. Abraxane is an oil-water emulsion designed to deliver paclitaxel, a chemotherapeutic agent [5]. This was the first nanomedicine FDA-approved for metastatic breast cancer [96].

### 7.4. Nanotechnology is Not Necessarily Progress

In light of decreasing funding for research around the world, pressure is on scientists to publish (sometimes) unsubstantiated claims to drum up public support, and thus attract more research funding. Hence there is some misinformation and uncalled-for hype surrounding nanomaterials, including COFs and MOFs. The much-lauded EPR effect, while fundamental to the action of many frameworks discussed in this review, is not unique to nanoparticles, for example [5]. An instance of similar hype in the 20th century was glucose-dependent insulin delivery, a type of 2nd-generation DDS, which ultimately proved ineffective [5].

### 7.5. Drug Delivery Systems: Targeting Problems

Key to effective nanoparticle therapeutics and diagnostics is proper targeting. Nanoparticles can increase drug concentration around a tumor by as much as 100% to 400% [5]. However, most of the administered drug migrates to nontarget sites, and some new DDS do not offer high enough clearance (removal from the target area). Despite these drawbacks, compensation arrives in the form of progressively higher drug loading in new materials, and up to five-fold more effective delivery, as illustrated by taxol, abraxane, genexol, and the like. Perhaps future work on COFs and MOFs for drug delivery will more often incorporate advanced targeting, such as specific antigen-based targeting.

### 7.6. Toxicity

Some nanomaterials—particularly MOFs—are inherently toxic. This poses a problem for nanomaterials intended for medicinal use. However, MOFs may be designed with “biologically benevolent” metals with lower toxicity such as Ca, Mg, Zn, Fe, Ti, and Zr [26], and can even be designed using endogenous linkers that the body can metabolize.

### 7.7. Economics and Looking Forward

Paramount to drug companies is the continuous improvement of technology that has already earned FDA approval [39]. Such technologies represent low risk and good financial return in the high-stakes world of drug discovery. Therefore, drug manufacturers may prefer spending their research, legal, and lobbying resources on modifications of existing technology instead of novel therapies. Hence medical applications of COFs and MOFs might take decades, if ever, to attain commercial viability.

## 8. Critical Assessment of the Field and Conclusions

Covalent and metal organic frameworks have considerable potential in biomedicine. These nanocarriers could herald a new era of targeted, stimulus-sensitive drug delivery, safe and sensitive imaging, and combined imaging and treatment. The ability to predict COF/MOF stability, drug loading, and other properties in silico is significant. The in vitro and in vivo effectiveness of COFs and MOFs that combine drug delivery with other disease-fighting techniques like photodynamic therapy and photothermal therapy is particularly impressive. However, at this stage in the development of COFs and MOFs, it would be unrealistic to pronounce them the next great advance in medicine. It is far too early to begin clinical trials on any COF or MOF-based treatment system, and pharmaceutical companies wisely choose to fund high-return research and trials; there is no shortage of nanomaterials research to be funded, for that matter. These companies may choose to pour their research and marketing resources into variations on old technology instead of gambling on unproven nanotechnologies. COFs and MOFs have inherent drawbacks that might preclude researchers from receiving well-deserved funding—COFs are often designed with toxic linkers, and MOFs with toxic metals. Still, significant progress has been made with the biologically-friendly MOFs mentioned in this article.

This review of Covalent Organic Frameworks and Metal Organic Frameworks hints at their encouraging future in biomedicine. Both types are crystalline and porous, they can be designed with custom properties, and they may be functionalized with post-synthetic modification—advantages not typically shared with other nanomaterials. COFs and MOFs have been demonstrated as effective drug delivery systems, agents for photothermal and photodynamic therapy, heavy metal adsorbents, antimicrobial agents, contrast and diagnostics agents, and more. They have shown promising results against various cancers and may be designed to specifically target cancer cells. However, creating crystalline, covalent extended structures like COFs remains fundamentally difficult, as does scaling the production of frameworks for commercialization. The exciting performance of MOFs in biomedical settings is tempered by the instability of some MOFs and the toxicity of many metal centers. Ultimately, any MOFs or COFs destined for the clinic must first meet stringent safety and efficacy standards set by regulatory agencies. Nevertheless, COFs and MOFs merit continued research attention if tomorrow’s physicians are to be given the tools they deserve.

## Figures and Tables

**Table 1:** Some reported COF and MOF Drug-Delivery Systems.

Framework (Type/Year)	Metal or Monomer	Linker	Drug	Loading Capacity	Time to Release Load (Aqueous)	Reference
PI-COF-4 PI-COF-5 (COF/2015)	Pyromellitic dianhydride (PMDA)	Tetrahedral 1,3,5,7-tetraaminoadamantane TAA (PI-COF-4) Tetra(4-aminophenyl) methane TAPM (PI-COF-5)	IBU	24 wt % (PI-COF-4) 20 wt % (PI-COF-5)	6 days	Fang [22]
PAF-6 (COF/2011)	Cyanuric chloride (CC)	biperazine	IBU	35 wt %	46 h	Zhao [11]
PMX@SOF (COF/2017)	Pyridinium-based tetracationic monomer (variable composition)	Cucurbit [8] uril ring	Pemetrexed	23 wt %	60 h	Tian [65]
TpASH TpAPH (COF/2017)	Tp	ASH APH	5-FU	12 wt %	72 h	Mitra [56]
5-FU@PI-2-COF (COF/2016)	1,3-5-triformyl benzene	4,4’-biphenyldiamine	5-FU	30 wt %	-	Bai [23]
DhaTab (COF/2015)	2,5-dihydroxyterephthalaldehyde	1,3,5-tris(4-aminophenyl) benzene	DOX	35 wt %	>7 days	Kandambeth [57]
TTI-COF (COF/2016)	Triazine triphenyl aldehyde (TT-ald)	Triazinetriphenylamine TT-am	Quercetin	-	-	Vyas [66]
NCTP (CTP/COF/2016)	Cyanuric chloride (CC)	Biphenyl	DOX	25 wt %	48 h	Rengaraj [24]
PCTF-Mn (CTP/Amorphous/2017)	5,10,15,20-tetraphenylporphyrin (TPP)	Cyanuric chloride (CC)	IBU	23 wt %	48 h	Luo [69]
HKUST-1(MOF/2007)	Cu (Cu-(NO_3_)_2_·3H_2_O)	Benzene1,3,5-tricarboxylic acid	NO	Approx. 3 mmol g^−1^	8 min	Xiao [87]
MIL-100 (MOF/2013)	Fe (III)	1,3,5-benzene tricarboxylic acid	Caffeine	49.5 wt %	48 h	Cunha [88,98]
UiO-66 (MOF/2013)	Zr (IV)	1,4-benzenedicarboxylate (BDC)	Caffeine	22.4 wt %	48 h	Cunha [88,99]
MIL-53 (MOF/2013)	Fe (III)	1,4-benzenedicarboxylate (BDC)	Caffeine	29.2 wt %	>216 h	Cunha [88,100]
MIL-127 (MOF/2013)	Fe (III)	3,3′,5,5′-azobenzenetetracarboxylate	Caffeine	15.9 wt %	48 h	Cunha [88,101]
NCP-1 (MOF/amorphous silica coating/2008)	Pt (IV)	c,c,t-diammine dichlorodisuccinate	Pt species	-	-	Rieter [102]
BioMIL-1 (MOF/2010)	Fe (III)	Pyridine-3-carboxylic acid (nicotinic acid)	Nicotinic acid	75 wt %	1 h	Miller [89]
UiO-68-type NMOF (MOF/hydrogel coating/2018)	Zr (IV)	Amino-triphenyl dicarboxylic acid	DOX	72.6 nmol mg^−1^	-	Chen [63]
NMOF (MOF/VEGF functionalized coating/2018)	Zr (IV)	Amino-triphenyl dicarboxylic acid	DOX	48.1 nmol mg^−1^	-	Chen [95]
ZIF-8 based (MOF/protein composite/2018)	Zn (II)	2-methylimidazole (Hmim)	DOX	10 wt %	24 h	Liang [96]
